# The Impact of Adjuvant Management Strategies on Outcomes in Women With Early Stage Uterine Serous Carcinoma

**DOI:** 10.7759/cureus.13505

**Published:** 2021-02-23

**Authors:** Andrew Cook, Remonda Khalil, Charlotte Burmeister, Irina Dimitrova, Mohamed A Elshaikh

**Affiliations:** 1 Radiation Oncology, Henry Ford Health System, Detroit, USA; 2 Biostatistics, Henry Ford Health System, Detroit, USA; 3 Gynecologic Oncology, Henry Ford Health System, Detroit, USA

**Keywords:** endometrial carcinoma, serous carcinoma, adjuvant radiation therapy, hysterectomy, hysterectomy, adjuvant chemotherapy

## Abstract

Objective

To determine the impact of different adjuvant strategies on outcomes in women with early-stage uterine serous carcinoma (USC).

Methods

Our retrospective database for women with endometrial carcinoma was queried for women with 2009 International Federation of Gynecology and Obstetrics (FIGO) stages I-II USC who underwent surgical staging between January 1991 and April 2019 followed by adjuvant management (observation, radiation therapy (RT), chemotherapy (CT), or combined modality treatment (CRT)). Chi-square tests were performed to compare differences in outcome by type of adjuvant management. Recurrence-free survival (RFS), disease-specific survival (DSS), and overall survival (OS) were assessed by Kaplan-Meier and log-rank tests. Univariate and multivariate analyses (MVA) were performed to identify statistically significant predictors of survival endpoints.

Results

We identified 171 women who met our inclusion criteria. The median follow-up time was 70.5 months. Seventy-five percent of the study cohort was FIGO stage IA, 13% were stage IB, and 12% were stage II. All women underwent pelvic lymph node dissection with a median number of dissected lymph nodes of 14. Omentectomy was performed in 64% of patients. Adjuvant RT was utilized in 56% of women (65 patients received vaginal brachytherapy alone, 10 patients received pelvic RT, and 21 patients received a combination of both). The most commonly used chemotherapy regimen was carboplatin and paclitaxel with a median number of cycles of six. A total of 44% of the cohort received CRT, 12% received RT alone, 19% received chemo alone, and 25% were observed. Five-year RFS was 73% for those who received CRT, 84% for those who received RT alone, 68% for those who received CT alone, and 55% for those who were observed (*p*=0.13). Five-year DSS was 81%, 94%, 71%, and 60%, respectively (*p*=0.02). Five-year OS was 76%, 70%, 60%, and 56%, respectively (*p*=0.11). On MVA of OS and DSS, a higher percentage of myometrial invasion, the presence of lower uterine segment involvement, positive peritoneal cytology, and receipt of chemotherapy alone/observation were independent predictors of worse outcomes. The sole independent predictor of worse RFS on MVA was the presence of positive peritoneal cytology.

Conclusion

In this cohort of women with early-stage USC who underwent surgical staging, adjuvant radiation treatment with or without chemotherapy was associated with improved survival endpoints and trended toward improved recurrence rates.

## Introduction

Endometrial cancer remains the most commonly diagnosed gynecologic malignancy in the United States [1]. Women with early-stage endometrial cancer have a favorable prognosis, with five-year survival rates of around 85% [2]; however, there remains a subset of 10% of patients who are diagnosed with uterine serous carcinoma (USC), which is known to have aggressive biology [3-4]. These patients have poorer outcomes in terms of recurrence and survival rates [5-10]. Although patients with USC comprise less than 15% of patients with endometrial carcinoma, they cause 40% of endometrial cancer deaths [11]. Furthermore, patients with early-stage USC constitute a heterogenous population with various risk factors. Studies for women with early-stage USC have found that deep myometrial invasion and positive peritoneal cytology are independent predictors of mortality [12-14]. Additional factors that may portend a poor prognosis for early USC are cervical stromal invasion and lymphovascular space invasion [15-16]. Outside of pathologic factors, African American patients with endometrial cancer have been found to have higher mortality rates [17].

Surgical staging is the cornerstone in the management of women with USC. Knowing that there is a high risk of recurrence in these patients, there is interest in optimizing adjuvant therapy. Given the low numbers of patients with early USC, it is difficult to accrue patients solely with USC histology to prospective randomized trials, and retrospective evidence has been necessary to guide treatment paradigms, albeit with conflicting reported outcomes.

While a recent study showed that adjuvant chemotherapy (chemo) with or without radiotherapy (RT) is associated with improved survival [18], several studies show that both chemo and RT are associated with improved survival outcomes compared to adjuvant chemo alone or RT alone in women with USC [19-21]. A large meta-analysis suggested a survival benefit to combined chemo and RT (CRT) for patients with early USC [22]. All of these adjuvant strategies, including observation, are valid approaches for early-stage USC according to the most recent National Comprehensive Cancer Network Uterine Neoplasms guidelines (version 1.2021).

These retrospective studies have been helpful in guiding adjuvant treatment; however, they are hampered with limitations that make them difficult to apply to clinical practice. These problems entail examining strictly non-myoinvasive USC [18], including advanced stages [22], and excluding patients who received pelvic RT [20]. Several studies also included patients with clear cell carcinoma [20-21,23]; clear cell carcinoma is thought to behave differently than USC and carries a different prognosis [24]. Additionally, some of these studies include analyses of large national databases that lack data on tumor recurrence [19].

Given that data remain conflicting on the optimal treatment for this subset of patients, the current study aimed to examine the outcomes of women with early-stage USC who underwent surgical staging followed by different adjuvant management approaches.

This research was earlier presented at the annual American Society for Radiation Oncology (ASTRO) meeting on October 24, 2020.

## Materials and methods

After institutional review board approval, our retrospective database was queried for patients with 2009 International Federation of Gynecology and Obstetrics (FIGO) stages I and II USC between the ages of 18 and 90 (age at hysterectomy). All patients were surgically staged between January 1991 and April 2017 and had at least one year of follow-up. Surgical staging was performed by a gynecologic oncologist with hysterectomy, salpingo-oophorectomy, with or without pelvic and para-aortic node dissection, and with or without omentectomy and peritoneal washings. These patients were divided into four groups based on adjuvant management received: observation, RT alone, chemo alone, or CRT. Patients with FIGO stage III-IV disease, non-serous endometrial histologies, synchronous malignancies, and non-operative candidates were excluded from this study.

Clinicopathologic factors that were evaluated in this study included patient age (as a continuous variable), body mass index (BMI), race, Charlson comorbidity score at the time of hysterectomy, percent myometrial invasion, FIGO stage, number of total lymph nodes (LN) examined (pelvic and paraaortic), peritoneal cytology status, lower uterine segment involvement, and lymphovascular space invasion (LVSI).

Survival curves were generated for the different adjuvant management groups using the Kaplan-Meier product-limit method calculated from the date of surgery. These were compared using the log-rank test to determine the rates of recurrence-free survival (RFS), disease-specific survival (DSS), and overall survival (OS). After univariate analysis, Cox regression analysis was performed to determine independent prognostic factors for RFS, DSS, and OS. Additionally, a backward selection process was used to restrict each of the models to contain all significant predictors. The Kruskal-Wallis and chi-square tests were used to determine significant differences (p < 0.05) in various clinical factors amongst the groups. All analyses were performed using SAS version 9.4 (SAS Institute, Inc., Cary, NC).

## Results

We identified 171 patients who met our inclusion and exclusion criteria. Median follow-up time was 70.5 months. None of the patients had residual disease post-hysterectomy. Thirty-five women (20%) were diagnosed with non-myoinvasive FIGO stage IA USC. There were 104 patients (61%) who underwent omentectomy as part of their surgical staging. Seventy-five patients (44%) received adjuvant CRT, 42 patients (25%) were followed with observation, 33 patients (19%) received adjuvant chemo alone, and 21 patients (12%) received adjuvant RT alone. For patients in the observation cohort, the reason why adjuvant therapy was not given was not clearly documented in the medical record; however, it is likely that most of these patients declined additional treatments. Table 1 summarizes information on patient characteristics amongst the groups, which were overall well-balanced. No statistically significant differences were noted between the groups except that a higher proportion of patients with FIGO stage IA disease were followed by observation after hysterectomy, and a higher proportion of patients in the CRT group underwent complete surgical staging with paraaortic lymph node dissection, omentectomy, and a higher median number of lymph nodes were examined.

**Table 1:** Patient Characteristics Amongst Treatment Groups Abbreviations: BMI, Body mass index; CRT, Chemoradiotherapy; Chemo, Chemotherapy; LN, Lymph node; LUS, Lower uterine segment; FIGO, International Federation of Gynecology and Obstetrics; RT, Radiation therapy; SD, Standard deviation

Variable	No Adjuvant Treatment (N=42)	RT Alone (N=21)	Chemo Alone (N=33)	CRT (N=75)	p
Age at hysterectomy, years, median (range)	67.5 (54-90)	71 (57-90)	68 (51-85)	66 (51-87)	0.26
Median BMI, kg/m^2^, median (range)	30.3 (19.6-54.3)	36.3 (17.9-48.7)	33.3 (22.5-64.1)	32.3 (18.3-52.8)	0.08
Race, n (%)					
White	21 (50%)	11 (52%)	17 (52%)	38 (51%)	0.97
African American	19 (45%)	10 (48%)	16 (48%)	35 (47%)	
Other	2 (5%)	0 (0%)	0 (0%)	2 (3%)	
2009 FIGO staging, n (%)					
IA	39 (93%)	11 (52%)	27 (82%)	51 (68%)	0.008
IB	3 (7%)	6 (29%)	2 (6%)	12 (16%)	
II	0 (0%)	4 (19%)	4 (12%)	12 (16%)	
LUS involvement, n (%)					
No	34 (81%)	12 (57%)	21 (64%)	53 (71%)	0.19
Yes	8 (19%)	9 (43%)	12 (36%)	22 (29%)	
Percent myometrial involvement, mean (SD)	10 (30)	40 (30)	20 (30)	30 (30)	0.01
LVSI					
Neg	32 (76%)	18 (86%)	30 (91%)	57 (76%)	0.26
Pos	10 (24%)	3 (14%)	3 (9%)	18 (24%)	
Dissection performed? n (%)					
No	7 (17%)	2 (10%)	4 (12%)	8 (11%)	0.81
Yes	35 (83%)	19 (90%)	29 (88%)	67 (89%)	
Number LN examined, median (range)	12.5 (0-72)	7 (0-64)	10 (0-45)	21 (0-56)	0.003
Number pelvic LN examined, median (range)	11.5 (0-37)	7 (0-36)	10 (0-38)	14 (0-31)	0.06
Number para-aortic LN examined, median (range)	0 (0-35)	0 (0-28)	0 (0-16)	5 (0-29)	<0.001
Para-aortic LN dissection performed? n (%)					
No	23 (55%)	15 (71%)	18 (55%)	19 (25%)	<0.001
Yes	19 (45%)	6 (29%)	15 (45%)	56 (75%)	
Type of surgery, n (%)					
Robotic	21 (50%)	3 (14%)	9 (27%)	37 (49%)	0.005
Open	21 (50%)	18 (86%)	24 (73%)	38 (51%)	
Omentectomy performed? n (%)					
No	15 (36%)	14 (67%)	14 (42%)	24 (32%)	0.03
Yes	27 (64%)	7 (33%)	19 (58%)	51 (68%)	
Type of RT					
External		5 (24%)		5 (7%)	<0.001
Brachy		3 (14%)		62 (83%)	
Both		13 (62%)		8 (11%)	
Peritoneal cytology, n (%)					
Negative	35 (83%)	18 (86%)	24 (73%)	60 (80%)	0.94
Positive	4 (10%)	2 (10%)	5 (15%)	8 (11%)	
Not done	3 (7%)	1 (5%)	4 (12%)	7 (9%)	
Recurrence, n (%)					
No	26 (62%)	17 (81%)	26 (79%)	58 (77%)	0.21
Yes	16 (38%)	4 (19%)	7 (21%)	17 (23%)	
Vaginal recurrence, n (%)					
No	34 (81%)	18 (86%)	29 (88%)	71 (95%)	0.17
Yes	8 (19%)	3 (14%)	4 (12%)	4 (5%)	
Pelvic recurrence, n (%)					
No	33 (79%)	21 (100%)	28 (85%)	61 (81%)	0.01
Yes	9 (21%)	0 (0%)	5 (15%)	14 (19%)	
Para-aortic recurrence, n (%)					
No	38 (90%)	21 (100%)	32 (97%)	66 (88%)	0.10
Yes	4 (10%)	0 (0%)	1 (3%)	9 (12%)	
Distant recurrence, n (%)					
No	33 (79%)	19 (90%)	29 (88%)	61 (81%)	0.25
Yes	9 (21%)	2 (10%)	4 (12%)	14 (19%)	
Vaginal only recurrence, n (%)					
No	39 (93%)	19 (90%)	32 (97%)	75 (100%)	0.17
Yes	3 (7%)	2 (10%)	1 (3%)	0 (0%)	
Pelvic only recurrence, n (%)					
No	41 (91%)	21 (100%)	33 (100%)	73 (97%)	0.14
Yes	1 (9%)	0 (0%)	0 (0%)	2 (3%)	
Vaginal/Pelvic recurrence, n (%)					
No	41 (98%)	21 (100%)	31 (94%)	75 (100%)	0.14
Yes	1 (2%)	0 (0%)	2 (6%)	0 (0%)	
Para-aortic only recurrence, n (%)					
No	40 (95%)	21 (100%)	33 (100%)	74 (99%)	1.00
Yes	2 (5%)	0 (0%)	0 (0%)	1 (1%)	

The most common adjuvant chemotherapy regimen used in the study cohort was carboplatin (median dose area under curve six) and paclitaxel (median dose 175 mg/m2) given every three weeks for three to six cycles (median six cycles). Adjuvant RT was given with either pelvic external beam RT (EBRT) alone, vaginal cuff high-dose-rate brachytherapy (VB) alone, or a combination of pelvic EBRT and VB.

VB was delivered using an iridium-192 source. If receiving VB alone, patients were given 30 Gy in five fractions prescribed to the surface of the upper four cm of the vagina. If combined with EBRT, the prescription was 18 Gy in three fractions to the same target. VB treatments were given with one to two fractions per week. Pelvic EBRT was delivered with either intensity-modulated RT or 3-dimensional RT technique to a median dose of 45 Gy (range 44-50.4 Gy), using 1.8 to 2.0 Gy per fraction with daily treatments over five to six weeks. All patients completed the prescribed course of RT without significant treatment interruptions. No patients who underwent RT experienced grade three or higher toxicity from RT.

**Figure 1:**
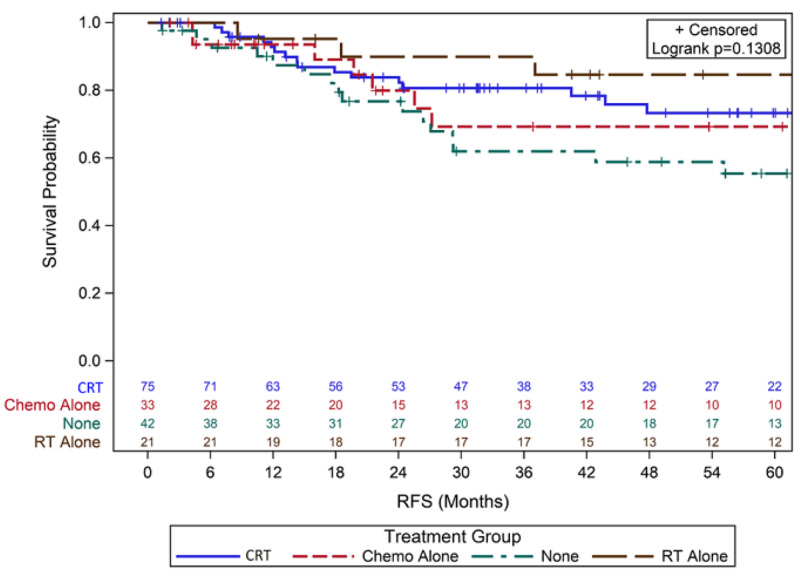
Kaplan-Meier Curves for Five-Year RFS Abbreviations: Chemo, Chemotherapy; RFS, Recurrence-free survival; RT, Radiation therapy

**Figure 2:**
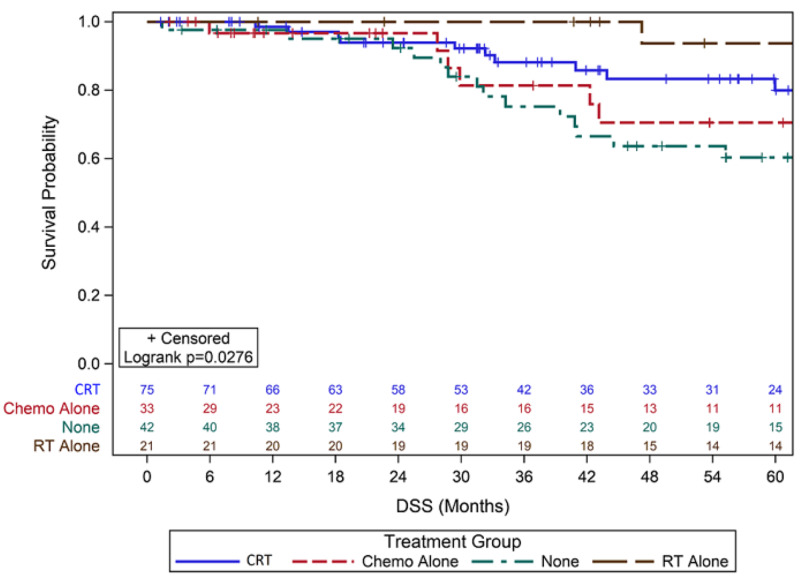
Kaplan-Meier Curves for Five-Year DSS Abbreviations: Chemo, Chemotherapy; DSS, Disease-specific survival; RT, Radiation therapy

**Figure 3:**
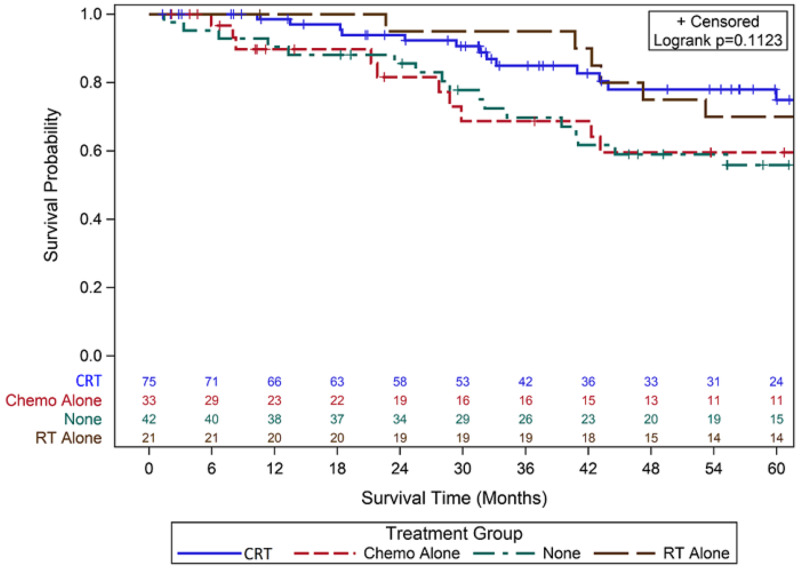
Kaplan-Meier Curves for Five-Year OS Abbreviations: Chemo, Chemotherapy; OS, Overall survival; RT, Radiation therapy

For overall recurrence, 44 women (26%) developed cancer recurrence as detailed in Table 1. Five-year RFS for those with no adjuvant treatment was 55% (95% Confidence Interval (CI) 37%-70%), 84% for RT alone (95% CI 58%-95%), 68% for chemo alone (95% CI 44%-84%), and 73% for CRT (95% CI 60%-83%) (p = 0.13) (Figure 1). Five-year DSS was 60% with observation (95% CI 42%-72%), 94% with RT alone (95% CI 66%-99%), 71% with chemo alone (95% CI 46%-86%), and 81% with CRT (95% CI 67%-90%) (p = 0.03) (Figure 2). Five-year OS was 56% with observation (95% CI 38%-70%), 70% with RT alone (95% CI 45%-85%), 60% for chemo alone (95% CI 38%-76%), and 76% for CRT (95% CI 61%-85%) (p = 0.114) (Figure 3).

Table 2 summarizes the variables included in the multivariate analysis (MVA) for RFS, DSS, and OS with their hazard ratios. The independent predictor of higher five-year RFS was negative peritoneal cytology. Independent predictors of higher five-year DSS included less myometrial invasion, negative peritoneal cytology, lack of lower uterine segment involvement, and the receipt of adjuvant CRT. Independent predictors of higher five-year OS were less myometrial invasion, lack of lower uterine segment involvement, and receipt of adjuvant CRT.

**Table 2:** Multivariate Analysis for Survival Endpoints for the Study Cohort Abbreviations: HR: Hazard ratio; CI, Confidence interval; LVSI, Lymphovascular space invasion; LUS, Lower uterine segment; CRT, Combined chemotherapy and radiation; RT, Radiation therapy; Chemo, chemotherapy; FIGO, International Federation of Gynecology and Obstetrics; N/A, Not applicable as it was not included in multivariate analysis

	Overall Survival			Recurrence-Free Survival			Disease-Specific Survival		
Variables	HR	95% CI of HR	p-value	HR	95% CI of HR	p-value	HR	95% CI of HR	p-value
Charlson Score	1.12	0.95-1.32	0.17	N/A	N/A	N/A	N/A	N/A	N/A
Percent Myometrial Invasion	10.09	2.84-35.88	<0.01	2.46	0.77-7.83	0.13	7.58	1.43-40.3	0.017
Cytology									
Negative vs Positive	0.47	0.22-1.01	0.052	0.44	0.20-0.96	0.04	0.37	0.15-0.92	0.03
LUS involvement	1.98	1.03-3.82	0.041	0.71	0.37-1.35	0.30	2.56	1.15-5.70	0.021
Negative LVSI	1.14	0.51-2.57	0.75	0.48	0.23-1.01	0.052	0.98	0.35-2.72	0.97
Treatment Group									
CRT vs RT	1.38	0.50-3.81	0.39	N/A	N/A	N/A	5.61	0.70-45.15	0.11
Chemo vs RT	3.00	1.01-8.94	0.049	N/A	N/A	N/A	10.1	1.15-88.7	0.04
Observation vs RT	5.20	1.73-15.62	0.003	N/A	N/A	N/A	22.8	2.66-195.31	0.004

## Discussion

As previously stated, the optimal adjuvant management for women with early USC remains controversial. Our study suggests that adjuvant RT with or without chemo provides statistically significant better uterine cancer-specific survival with a trend toward improving RFS and OS when compared to chemo alone and observation. This finding is in agreement with other studies that also showed improvement in survival outcomes with either RT alone or CRT [19-22,25-26]. Among the treatment groups in the DSS analysis, patients who received CRT and RT alone enjoyed the best five-year survival outcomes (81% and 94%, respectively). Additionally, our survival outcomes in patients who received RT as part of their treatment course are comparable to other prospective and retrospective studies [16,25-26].

As shown in our analysis, chemo alone was not found to be a significant predictor of improved OS, RFS, or DSS. This result has also been found in other studies, including a subset analysis of a randomized trial comparing adjuvant RT with or without sequential chemo. The results showed that chemo did not have an effect on progression-free survival or OS in patients with any stage serous or clear cell carcinoma [27]. Additionally, a study by Barney et al. looked at outcomes of early-stage serous and clear cell patients who received VB and showed that chemo did not improve survival outcomes [28].

Our study findings are also in agreement with the Gynecologic Oncology Group-249 (GOG-249) study, a large randomized trial that compared pelvic RT alone versus VB plus three cycles of chemo in women with stage I-II endometrial carcinoma with endometrioid and non-endometrioid histologies. It showed no significant difference in terms of survival outcomes in those who received three cycles of chemo with VB compared to those who received pelvic RT alone, although the proportion of women with USC in this study was small [29].

Our results contrast with those seen in the Post-Operative Radiation Therapy in Endometrial Cancer-3 (PORTEC-3) study, which showed that CRT improved survival outcomes compared to RT alone for early-stage USC, suggesting that chemo plays an important role in USC. However, PORTEC-3 included women with advanced-stage USC and excluded women with non-myoinvasive FIGO stage IA disease [24]. Twenty percent of our study cohort had a non-myoinvasive disease, and 75% of our patients were FIGO stage IA. It is also possible that our study is underpowered to detect a positive impact of adjuvant chemo on survival endpoints.

A limitation of this study is the selection bias inherent to a retrospective analysis, which may explain why some of our findings differ from prospective studies. Although the sample size of this study is relatively small compared to some of the larger prospective trials that have been mentioned, this analysis is one of the largest single-institution studies to date that examines outcomes solely in stage I-II USC. This analysis was able to confirm serous histology by an expert gynecologic pathologist in a respectable sample size. Furthermore, the study groups were overall well-balanced, which strengthens the findings of this study.

## Conclusions

The optimal adjuvant management strategy for early-stage USC remains controversial. This single-institution retrospective study evaluating the impact of different adjuvant management strategies on early-stage USC suggests that adjuvant RT with or without chemo improved DSS with a trend towards improved OS and RFS. Predictors of improved DSS were less myometrial invasion, negative peritoneal cytology, lack of lower uterine segment involvement, and receipt of adjuvant CRT. Further prospective trials will be needed to determine the optimal combination of chemo and RT for patients with this relatively rare histology.
